# Retraction Note: WELL.ME - Wellbeing therapy based on real-time personalized mobile architecture, vs. cognitive therapy, to reduce psychological distress and promote healthy lifestyle in cardiovascular disease patients: study protocol for a randomized controlled trial

**DOI:** 10.1186/s13063-018-3086-5

**Published:** 2018-12-20

**Authors:** Angelo Compare, Vassilis Kouloulias, Vontas Apostolos, Wendy Moreno Peña, Enrico Molinari, Enzo Grossi, Efstathopoulos Efstathios, Michele Carenini

**Affiliations:** 1 Department of Human and Social Sciences, Human Factors and Technologies for Health - HTC Centre, University of Bergamo, Piazzale S. Agostino 2, 24129 Bergamo, BG Italy; 2 Medical School, National University of Athens, Athens, Greece; 3 Technopolis ICT Business Park, Thessaloniki, Greece; 4 SOROS GABINETE, Socia ProgramasEuropeos, Europe, Spain; 5 Istituto Auxologico Italiano, Catholic University of Milan, Milan, Italy; 6 Medical Department, Bracco SpA, IULM – University, Fondazione Bracco, Milan, Italy; 7 NoemaLife Spa, Bologna, Italy

**Retraction Note: Trials**

**https://doi.org/10.1186/1745-6215-13-157**

The Editors-in-Chief are retracting this article [1]. After publication concerns were raised with respect to inconsistencies between this trial protocol and the trial registry entry. Subsequent investigation has shown that patient recruitment had been completed before submission of the protocol to the journal, which is a breach of the journal’s editorial policy. Concerns have also been raised that one of the reviewers was added to this article as an author after revision. Michele Carenini and Wendy Moreno Peña agree with this retraction and Angelo Compare does not agree with this retraction. Vassilis Kouloulias, Vontas Apostolos, Enrico Molinari, Enzo Grossi and Efstathopoulos Efstathios did not reply to correspondence about this retraction.

## References

[1] Compare A (2012). WELL.ME - Wellbeing therapy based on real-time personalized mobile architecture, vs. cognitive therapy, to reduce psychological distress and promote healthy lifestyle in cardiovascular disease patients: study protocol for a randomized controlled trial. Trials.

